# Association of the Kidney Failure Risk Equation With High Health Care Costs

**DOI:** 10.1016/j.ekir.2023.03.008

**Published:** 2023-03-20

**Authors:** Nancy L. Reaven, Susan E. Funk, Vandana Mathur, Thomas W. Ferguson, Julie Lai, Navdeep Tangri

**Affiliations:** 1 Strategic Health Resources, La Cañada, California, USA; 2 MathurConsulting LLC, Woodside, California, USA; 3 Department of Internal Medicine, Max Rady College of Medicine, University of Manitoba, Winnipeg, Manitoba, Canada; 4 Seven Oaks Hospital Chronic Disease Innovation Center, Winnipeg, Manitoba, Canada

**Keywords:** chronic kidney disease, health care costs, health economics, kidney failure, kidney failure risk equation, retrospective

## Abstract

**Introduction:**

The Kidney Failure Risk Equations (KFRE) are accurate and validated to predict the risk of kidney failure in individuals with chronic kidney disease (CKD), but their potential to predict health care costs in the US health care system is unknown. We assessed the association of kidney failure risk from the 4-variable and 8-variable 2-year KFRE models with monthly health care costs in US patients with CKD stages G3 and G4.

**Methods:**

This was an ancillary study to a larger observational, retrospective cohort study examining the association between serum bicarbonate and adverse kidney outcomes. Monthly medical costs were calculated from individual health care insurance claims. Generalized linear regression models were used to examine the association of KFRE score with health care costs.

**Results:**

A total of 1721 patients qualified for the study (1475 and 246 with CKD stages G3 and G4, respectively). For 8-variable KFRE, each 1% (absolute) increase in risk was associated with 13.5% (*P* < 0.001) and 4.1% (*P* < 0.001) higher monthly costs for patients with CKD stage G3 and G4, respectively. For 4-variable KFRE, a 1% increase in risk was associated with 6.7% (*P* = 0.016) and 2.9% (*P*= 0.014) increase in monthly costs for patients with CKD stage G3 and G4, respectively.

**Conclusion:**

Higher risks of kidney failure as predicted by the 4-variable or 8-variable KFRE were associated with higher 2-year medical costs for patients with CKD stages G3 and G4. The KFRE may be a useful tool to anticipate medical costs and target cost-reducing interventions for patients at risk of kidney failure.

## Graphical abstract

**Figure:**
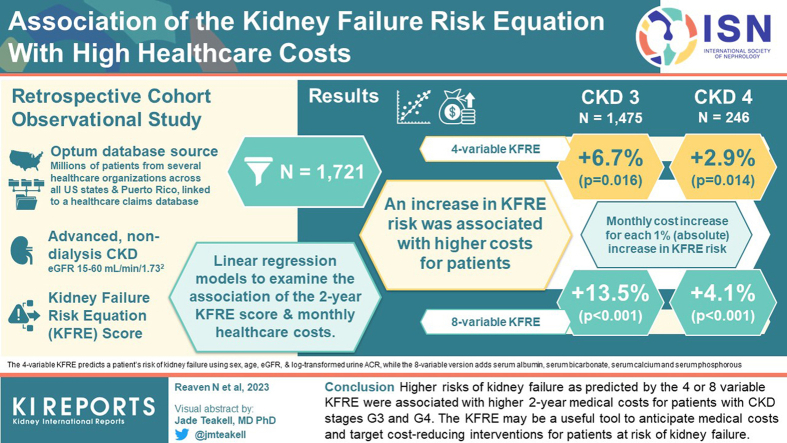

CKD is a common health condition that affects 9.1% of the global population.^1^^,^^2^ CKD is associated with increased hospitalizations, emergency department utilization, and all-cause mortality as well as decreased physical function.^3^, ^4^, ^5^, ^6^ CKD and its complications are associated with increasing costs as the disease progresses, reaching over $75,000 annually in the United States for patients with CKD stages G4 and G5 with commercial insurance coverage and over $35,000 annually for those receiving Medicare.^7^^,^^8^ Moreover, patients who progress to kidney failure experience very high health care costs, totaling over $50 billion annually for Medicare-related expenditures alone, accounting for over 7% of total Medicare expenditures, despite representing only approximately 800,000 individuals in the US.^8^

Recently, the Center for Medicare and Medicaid Services has suggested the introduction of care models with the aim of improving outcomes and reducing costs in CKD care. The program, the Advancing American Kidney Health executive order has 3 goals that are as follows: (i) reducing kidney failure risk, (ii) improving access and quality of treatment options, and (iii) increasing optimal kidney failure transition (focus on transplantation and home dialysis).^9^ This initiative will permit nephrologists and nephrology practices to assume various levels of financial risk for patients under their care under the Comprehensive Kidney Care Contracting reimbursement options or the Kidney Care First option, which provides capitation payments for managing care of patients with CKD stages G4 and G5 and on dialysis, with adjustment of these payments based on health outcomes adjusted to national standards.^10^

Tools to classify the risk of progression of patients with CKD can help to align the goals in the aforementioned care models. One such tool, the KFRE, is an internationally validated prediction tool that estimates the risk of progression to kidney failure and is highly accurate for prediction of kidney failure at 2 years and 5 years.^11^^,^^12^ The equation is used to determine referral to nephrology and multidisciplinary care,^13^^,^^14^ planning for dialysis modality education and deciding when to place hemodialysis access. ^15^^,^^16^

In a Canadian study, patients classified as high-risk of progression with the KFRE (5-year risk >15%) have been demonstrated to have approximately twice the costs of hospital admissions and physician visits and a 30% increase in drug dispensation expenses over a 5-year period compared with the low-risk group.^17^ Risk as determined by the KFRE and its association with costs in the US health care system, as well as a broader general population not referred for nephrology care, have not been studied. Therefore, we aimed to determine the relationship between risk as classified by the KFRE and all-cause medical costs from the perspective of the US health system payer.

## Methods

### Study Design and Data Sources

This observational, retrospective cohort study of US patients with advanced nondialysis-dependent CKD was conducted using Optum’s deidentified Integrated Claims-Clinical data set of US patients (2007–2017). The Optum database is a longitudinal repository of electronic health record (EHR) data that included >81 million patients as of 2017 from all insurance types or statuses and a subset of patients linked by unique patient identifiers to a health care claims database of private and Medicare Advantage health care insurance plans.^18^ The Optum database includes patients from several health care provider organizations across all 50 US states and Puerto Rico. Data extracted from inpatient and outpatient EHRs and administrative systems included laboratory results, prescribed medications, coded diagnoses, and procedures as well as provider notes extracted by natural language processing. Institutional review board oversight was not required because the Optum EHR Database contained only Health Insurance Portability and Accountability Act-compliant, deidentified data. Data cleaning is described in Supplementary Table S1.

### Study Cohort

**Figure 1:**
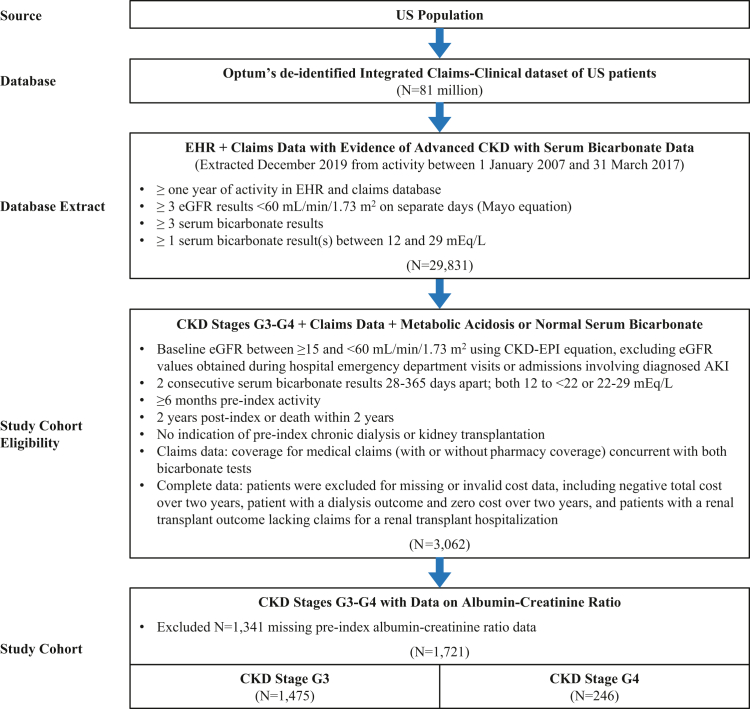
Study cohort selection diagram. AKI, acute kidney injury; CKD, chronic kidney disease; CKD-EPI, Chronic Kidney Disease Epidemiology Collaboration; eGFR, estimated glomerular filtration rate; EHR, electronic health record.

This was an ancillary study of a larger observational, retrospective study to determine the association of serum bicarbonate levels with clinical outcomes. Patients included in the database extract with claims data had at least 1 year of EHR activity with at least 3 estimated glomerular filtration rate (eGFR) results <60 ml/min per 1.73 m^2^ and at least 3 serum bicarbonate results, with at least 1 result between 12 and 29 mEq/l, plus linked claims data (Figure 1). To qualify for the study cohort, patients were required to have 2 consecutive serum bicarbonate results 28 to 365 days apart in the EHR data, both in the range of 12 to <22 mEq/l (metabolic acidosis group) or 22 to 29 mEq/l (normal serum bicarbonate group), and claims data concurrent with both bicarbonate tests. The first value in each pair established baseline serum bicarbonate and its test date established the index date. Inclusion also required a baseline eGFR value >15 and <60 ml/min per 1.73 m^2^, calculated as the mean of eGFR values for 90 days preceding the last eGFR test before or on the index date. eGFR values estimated during hospital inpatient admissions or emergency department visits with diagnosed acute kidney injury were excluded because they could represent acute events. Study inclusion also required EHR activity for ≥6 months preindex plus ≥2 years postindex unless the patient died within this 2-year period. Patients with preindex evidence of chronic dialysis or kidney transplantation (by diagnosis or procedure code or any outpatient eGFR ≤10 ml/min per 1.73 m^2^) were excluded, so were patients’ missing data on baseline albumin-to-creatinine ratio (ACR). Patients were classified by baseline CKD stages G3 or G4 using baseline eGFR. Definition details including code lists are provided in Supplementary Table S1.

To ensure sufficient representation at low serum bicarbonate levels, an iterative selection algorithm was used to oversample the number of patients with metabolic acidosis by initially selecting qualifying pairs of serum bicarbonate in the 12 to <22 mEq/l range before examining bicarbonate values between 22 and 29 mEq/l. This methodology is further explained in a previous publication, along with sensitivity analyses showing that primary clinical findings were sustained in the absence of oversampling.^19^

### Variables

The primary variable of interest was the predicted risk of kidney failure within 2 years, calculated for each patient using the 8-variable and 4-variable versions of the KFRE.^11^^,^^12^ The 4-variable KFRE predicts a patient’s risk of kidney failure using sex, age, eGFR, and log-transformed urine ACR, whereas the 8-variable version adds serum albumin, serum bicarbonate, serum calcium, and serum phosphorous (Supplementary Table S2). In this study, serum bicarbonate and eGFR were evaluated at baseline, with the risk score calculated at an index date determined by the first of 2 serum bicarbonate tests used to determine metabolic acidosis as described in the cohort selection above. Urine ACR was evaluated using the value reported for an available urine ACR or value converted from a urine protein-to-creatinine ratio or dipstick urine test^20^^,^^21^ that was evaluated on the index date or as the closest value up to 1 year before the index date. Corrected calcium was calculated using baseline serum albumin if available, otherwise unadjusted total calcium was used. Data on calcium, phosphorous, and albumin was limited to values occurring within a year on or before the index date.

Additional descriptive statistics were provided for sex and race, as well as diabetes and hypertension, which were identified by single occurrence of an International Classification of Diseases-9 and 10-Clinical Modification diagnosis code in all available preindex data, excluding codes with a status of “possible” or “indicative of personal or family history”.

Serum calcium was missing in 5% of patients in CKD stage 3 and 0% of patients in CKD stage 4, and serum albumin was missing in 21% of patients in CKD stage 3 and 13% of patients in CKD stage 4. Missing data for serum calcium and serum albumin were imputed 10 times by multiple imputation using the Markov chain Monte Carlo method and reported at the mean of 10 imputations in descriptive results.^22^ Serum phosphorous was omitted from the 8-variable KFRE equation because of a high proportion of missing data in this cohort. Additional information on variable definitions, including date and validity parameters, data sources, and conversions is provided in Supplementary Table S1.

### Outcome

The outcome was all-cause medical costs per month, assessed during the 2-year outcome period. Outpatient pharmacy costs were not included in this analysis. Medical costs in the Optum data set were obtained from medical insurance claims for inpatient and outpatient services of all types and provided from the payer perspective (i.e., health plan payments rather than provider-billed charges) as Optum-defined “standard cost,” an approximated cost per service based on private insurance payment levels in 2015 US dollars. This methodology accounts for service quantity, relative resource costs, and the nature of the service rendered to reduce variation in payment amounts to similar providers for the same service. For inpatient confinements missing cost data, costs were imputed at the average per-diem rate for other admissions for the same patient or (for 3 patients) at the per-diem mean of the total cohort. Costs were inflated to 2021 US dollars at 3% per annum.

Medical cost per month was calculated as the 2-year total of costs for inpatient and outpatient medical claims, divided by the number of months in the observation period (24 months, unless shortened by death). To address typical skew in health care costs, patient-level medical cost per month was adjusted with a 98% winsorization, setting all results less than the first percentile of medical cost per month to zero and all costs greater than the 99th percentile of medical cost per month to that 99th percentile.^23^ Cost per month was log-transformed in the current analysis to further address skew.^24^

### Statistical Analysis

Patient characteristics were reported at mean values with standard deviation or as percentages. Results for imputed laboratory variables were reported at the mean of 10 imputations. For descriptive analyses, patients in each CKD stage (G3 and G4), and overall, were ranked highest to lowest in risk of kidney failure by the 8-variable KFRE and assigned to risk quartiles within stage. Average cost per month in 2021 US dollars was calculated by risk quartile within each stage and evaluated pairwise between risk quartiles by a generalized linear model comparison of least squares means.

Generalized linear models were run within CKD stage G3 and stage G4 groups, equating log-transformed medical cost per month with the calculated risk score with both the 8-variable and 4-variable KFREs. Because the 8-variable KFRE risk score models contained missing serum calcium and serum albumin data, multiple imputation was implemented 10 times to generate 10 complete datasets. These 10 complete datasets were then analyzed separately using the 8-variable KFRE risk score as the sole predictor of medical cost per month (log-transformed). The results of the 10 analyses were combined in the SAS PROC MIANALYZE procedure to derive valid inferences. Similar sets of generalized linear model analyses were run on models that evaluated each component of the 4-variable and 8-variable KFRE equations as individual covariates as follows: (i) a model with age, sex, eGFR, and log-transformed urine ACR (4 variable) and (ii) a model with age, sex, eGFR, log-transformed urine ACR, serum bicarbonate, serum albumin, and serum calcium (8 variable, excluding serum phosphorous).

All statistical analyses were performed using SAS/STAT software, version 9.2 (Cary NC, USA). *P*-values <0.05 were considered statistically significant.

## Results

The Optum database contained longitudinal records of approximately 81 million patients, of which 29,831 met the criteria for inclusion in the database extract with claims. Within this extract, 1721 patients qualified for the study cohort as follows: 1475 in CKD stage G3 and 246 in CKD stage G4 (Figure 1).

Mean (standard deviation) age was 75.3 (9.8) years in the CKD stage G3 group and 76.2 (9.1) years in the CKD stage G4 group (Table 1). The CKD stage G3 and G4 groups were 49% and 46% male, 88% and 85% White race, respectively. Mean (standard deviation) baseline serum bicarbonate was 25.5 (4.3) mEq/l in patients with CKD stage G3 and 23.2 (4.6) mEq/l in the CKD stage G4, whereas mean (standard deviation) serum albumin was 3.8 (0.6) g/dl and 3.6 (0.6) g/dl, respectively. Diabetes was identified in 39% of the CKD stage G3 group and 50% of the CKD stage G4 group. Hypertension was highly prevalent in both groups; 73% in CKD stage G3 and 80% in CKD stage G4. Kidney failure risk scores using the 4-variable and 8-variable KFRE equations showed an average predicted risk of kidney failure within the next 2 years of 0.8% to 1.0% in the CKD stage G3 group and 8.6% to 9.2% in the CKD stage G4 group, respectively.

**Table 1:** Baseline characteristics

Characteristic	CKD stage G3 (*N* = 1475)	CKD stage G4 (*N* = 246)
Age (yr), mean ± SD	75.3 ± 9.8	76.2 ± 9.1
Sex (male), *n* (%)	717 (49)	112 (46)
Race, *n* (%)		
Asian	20 (1)	4 (2)
Black	101 (7)	25 (10)
White	1292 (88)	210 (85)
Other/Unknown Race	62 (4)	7 (3)
Baseline comorbidities, *n* (%)		
Diabetes	572 (39)	123 (50)
Hypertension	1082 (73)	197 (80)
Baseline laboratory values, mean ± SD		
Serum bicarbonate (mEq/l)	25.5 ± 4.3	23.2 ± 4.6
eGFR (ml/min per 1.73 m^2^)	45.6 ± 8.3	23.9 ± 4.1
Urinary albumin-creatinine ratio (mg/g)	131 ± 513	275 ± 696
Serum calcium (mg/dl)	9.3 ± 0.6	9.4 ± 0.7
Serum albumin (g/dl)	3.8 ± 0.6	3.6 ± 0.6
2-yr KFRE (8-variable) score (%), mean ± SD^a^	1.0 ± 1.9	9.2 ± 9.7
2-yr KFRE (4-variable) score (%), mean ± SD	0.8 ± 1.5	8.6 ± 10.2

CKD, chronic kidney disease; eGFR, estimated glomerular filtration rate; KFRE, kidney failure risk equation.

a For the 8-variable KFRE equation, serum calcium and serum albumin were imputed where missing, serum phosphorous was imputed for all patients at the equation coefficient, effectively omitted from calculation because of missing data in a substantial majority of patients.

**Figure 2:**
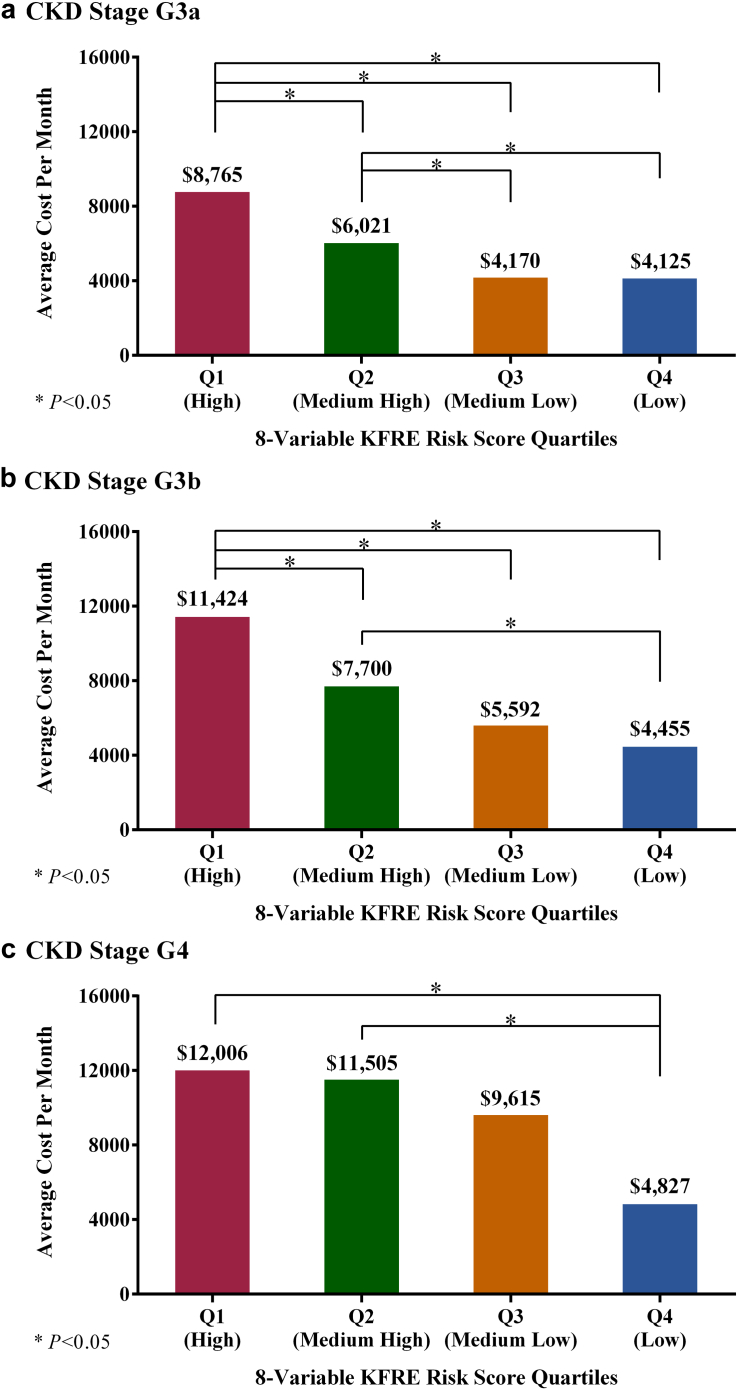
Average cost per month by risk quartile and CKD stage. CKD, chronic kidney disease; KFRE, kidney failure risk equation.

**Figure 3:**
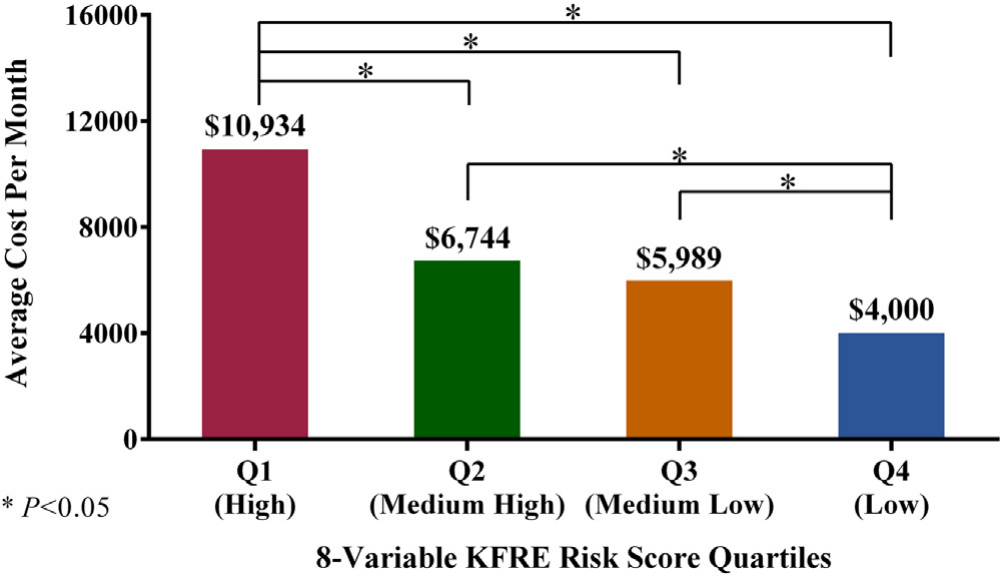
Average cost per month by risk quartile. KFRE, kidney failure risk equation.

Higher levels of kidney failure risk correlated with higher costs in every stage of CKD. Patients in the highest quartile of kidney failure risk as measured by the 8-variable KFRE had average costs per month over a 2-year outcome period of $8765 in stage G3a, $11,424 in stage G3b, and $12,006 in stage G4, versus $6021 (*P* = 0.003), $7700 (*P* = 0.002), and $11,505 (*P* = 0.8) for patients in the second-highest quartile of kidney failure risk, respectively (Figure 2). More advanced CKD was consistently associated with higher costs. Average cost per month was approximately double between the lowest and highest quartile for patients with CKD stage G3a ($8765 vs. $4125), and approximately 2.5-fold to 3-fold higher between the lowest and highest quartile in patients with CKD stage G3b ($11,424 vs. $4455) and in patients with CKD stage G4 ($12,006 vs. $4827). In all CKD stages, costs were statistically similar between the 2 lowest quartiles of kidney failure risk (Figure 2). When considering the entire population not stratified into CKD stages, patients in the highest quartile of risk had a monthly cost of $10,934, over 2.5-fold higher than those in the lowest risk quartile with a monthly cost of $4000. Those in the middle quartiles were not statistically different from each other with monthly costs ranging from $5989 to $6744 (Figure 3).

The 8-variable KFRE was found to be significantly associated with health care costs in patients with CKD stages G3 and G4. Adjusted analyses indicated that each 1% (absolute) increase in the predicted risk score was associated with a 13.5% (parameter estimate 0.126, 95% confidence interval: 0.085–0.167; *P* < 0.0001) and 4.1% (parameter estimate 0.040, 95% confidence interval: 0.022–0.058; *P* < 0.0001) increase in predicted monthly health care costs in patients with CKD stages G3 and G4, respectively (Table 2). The fully specified model in patients with CKD stage G3 found the following 2 variables to be significant predictors of monthly costs: (i) serum bicarbonate (1 mEq/l increase associated with a 7.0% decrease in monthly costs) and (ii) serum albumin (1 g/dl increase associated with a 55.5% decrease in monthly costs). These findings were similar among patients with CKD stage G4 as follows: (i) serum bicarbonate (1 mEq/l increase associated with a 4.6% decrease in monthly costs) and (ii) serum albumin (1 g/dl increase associated with a 52.1% decrease in monthly costs).

**Table 2:** Generalized linear regression models on impact of 2-yr kidney failure risk and the component of KFRE independently on log all-cause cost per month

Covariate	CKD stage G3		CKD stage G4	
	Estimate (95% CI)	*P-v*alue	Estimate (95% CI)	*P-v*alue
8-variable kidney failure risk equation				
Model 1: kidney failure risk score only				
2 yr kidney failure risk	0.126 (0.085, 0.167)	<0.0001	0.040 (0.022, 0.058)	<0.0001
R-squared (mean of 10 imputations)	0.024		0.071	
Model 2: component of KFRE independently				
Serum bicarbonate, per 1 mEq/l increase	−0.073 (−0.091, −0.055)	<0.0001	−0.047 (−0.086, −0.009)	0.0167
Age, per 1 yr increase	0.000 (−0.007, 0.008)	0.9041	−0.015 (−0.034, 0.005)	0.1429
eGFR, per 1 ml/min per 1.73 m^2^ increase	0.001 (−0.008, 0.009)	0.9001	−0.029 (−0.072, 0.013)	0.1754
Serum calcium, per 1 mg/dl increase	−0.045 (−0.177, 0.088)	0.5073	0.073 (−0.193, 0.338)	0.5915
Male, vs. female	−0.045 (−0.190, 0.100)	0.5427	0.166 (−0.192, 0.524)	0.3630
Log urine albumin-creatinine ratio, per 1-unit increase	0.015 (−0.034, 0.064)	0.5546	0.053 (−0.051, 0.156)	0.3176
Serum albumin, per 1 g/dl increase	−0.809 (−0.964, −0.653)	<0.0001	−0.737 (−1.050, −0.424)	<0.0001
R-squared (mean of 10 imputations)	0.166		0.164	
4-variable kidney failure risk equation				
Model 1: kidney failure risk score only				
2 yr kidney failure risk	0.065 (0.012, 0.118)	0.0157	0.029 (0.011, 0.046)	0.0014
R-squared (mean of 10 imputations)	0.004		0.041	
Model 2: component of KFRE independently				
Age, per 1 yr increase	−0.002 (−0.010, 0.006)	0.6382	−0.013 (−0.034, 0.008)	0.2198
eGFR, per 1 ml/min per 1.73 m^2^ increase	−0.007 (−0.016, 0.002)	0.1486	−0.030 (−0.075, 0.015)	0.1886
Male, vs. female	−0.061 (−0.217, 0.095)	0.4432	0.100 (−0.266, 0.466)	0.5939
Log urine albumin-creatinine ratio, per 1 unit increase	0.087 (0.035, 0.139)	0.0011	0.083 (−0.025, 0.191)	0.1323
R-squared (mean of 10 imputations)	0.010		0.036	

CI, confidence interval; CKD, chronic kidney disease; eGFR, estimated glomerular filtration rate; KFRE, kidney failure risk equation;

R-squared for each model is reported as the mean result of 10 imputations using SAS PROC MIANALYZE.

For the 4-variable KFRE, each 1% increase in the predicted risk score was significantly associated with a 6.7% (parameter estimate 0.065, 95% confidence interval: 0.012–0.118; *P* = 0.0157) and 2.9% (parameter estimate 0.029, 95% confidence interval: 0.011–0.046; *P* = 0.014) increase in monthly medical costs in patients with CKD stages G3 and G4, respectively (Table 2). With respect to the fully specified model, in patients with CKD stage G3, only urine ACR was a significant predictor (a doubling of urine ACR in mg/g is associated with a 6.2% increase in monthly costs) and among patients with CKD stage G4, no individual variables were significantly associated individually.

A summary of results for the generalized linear models is provided in Table 2.

## Discussion

In this study of patients with CKD stages G3 and G4, we found that higher risk of progression to kidney failure as calculated by the KFRE was associated with higher monthly all-cause medical costs in a population-based sample of US patients. These findings are consistent with previous Canadian data associating KFRE-based risk to health care costs,^17^ and extend these observations to a larger data set in the US health care system. Because nephrology practices integrate the KFRE in their EHRs or clinical decision support software, they can assume that the highest risk individuals as identified by the equation are also likely to have the highest health care costs.

To our knowledge, this analysis is the first to evaluate the association of the risk of CKD progression as measured by the KFRE and associated health care costs in a US population. A previous study was performed in Canada among patients referred to a multidisciplinary care CKD clinic that examined 5-year costs and their association with risk of CKD progression as measured by the KFRE and found that the total annual cost of hospital admissions, physician visits, and drug dispensations totaled over $134,000 for patients at high risk of progression (>15% risk of failure over 5 years) versus approximately $76,000 for those at low risk of progression (Canadian dollars).^17^ Our study differed from the Canadian study because it was done in a US population and included all identified patients with CKD in Optum rather than those enrolled in a multidisciplinary clinic with subjective enrollment criteria. A previous study of private and Medicare patients in the US found the annual cost of nondialysis CKD care to range between $26,000 and $77,000 for patients with CKD stages G3a to G5 with private insurance coverage and between $21,000 and $46,000 for patients receiving Medicare (2016 US dollars).^7^

The primary components associated with cost from the 8-variable KFRE were serum bicarbonate and serum albumin. Each of these variables, when modified by 1 standard deviation from their means, represented a 30% change in estimated monthly costs. These biomarkers likely affect costs because of their association with adverse outcomes. Serum albumin is a marker of nutritional status and has been shown to be associated with physical function decline and failure to thrive. ^25^^,^^26^ Reductions in serum bicarbonate are associated with progression of kidney disease,^27^ cardiovascular events,^28^ and acute kidney injury.^29^ In our analyses examining the 4-variable KFRE, albuminuria was most strongly associated with higher cost, though the effect of eGFR on costs may have been limited by the restriction of the eGFR range by CKD stage.

This study had several strengths. It analyzed costs from a large population health repository and therefore improves upon previous studies examining cost by risk that were limited to patients enrolled in multidisciplinary CKD care and is likely generalizable to a wider population of patients with CKD. It also further builds on the evidence for the utility of the KFRE in managing CKD care in the US health system, providing additional evidence that risk scores predicting progression of CKD are associated with increase in health-related expenses.

This analysis also had several limitations. First, the cost data was obtained from patients with 2 types of common insurance (private and Medicare Advantage) and may not be representative of other payment types (e.g., Medicaid). Urine ACR and serum bicarbonate were required for inclusion in the study for assessment of the KFRE (measured directly or by transforming a urine protein-to-creatinine ratio/urine dipstick result), and therefore may not be representative of patients in whom no such measurement was available. Although these findings confirm a previous study in the Canadian health system,^17^ they may not reflect implications, risks, and costs in other countries; these country-specific analyses may require future evaluation. In addition, as outpatient pharmacy costs were not available in the Optum data set, we were unable to include them in our results. Lastly, the KFREs do not account for other variables such as other concomitant medical conditions (e.g., malignancy) or aspects associated with frailty and disability, which may be reflected in the low R-squared value observed in the models, and alternative scores may be required if the goal is to optimize the prediction of health care costs.

In conclusion, higher risk of progression to kidney failure as measured by the KFRE is associated with higher all-cause health care costs in patients with private insurance and Medicare Advantage in the United States. Use of risk stratification equations to identify patients at the highest risk of progression and subsequent use of health care resources may help to effectively target resources.

## Disclosure

NLR, SEF, VM, TWF, JL, and NT were paid consultants to Tricida, Inc. in connection with the development of this manuscript. NLR, VM, and NT report consultancy, personal fees, and equity ownership from Tricida, Inc., related to the submitted work. SEF and TWF report consultancy and personal fees from Tricida, Inc. VM and NT are members of advisory boards at Tricida. VM is listed on patents related to work for Tricida. VM reports additional consulting fees from Tricida, Equillium, Myovant, Rigel, Corvidia, Acuta, Frazier, Intarcia, PTC Bio, Escient, RallyBio, Unicycive, Galderma, and Sanifit outside the submitted work.

## Supplementary Material

Supplementary File (PDF)

**Table S1.** Sources and definitions.

**Table S2.** Kidney failure risk equation.

**STROBE statement.**
