# Supplementary material for: Association of the Kidney Failure Risk Equation With High Health Care Costs
Source: Kidney Int Rep. 2023 Mar 20;8(6):1183–91. doi: 10.1016/j.ekir.2023.03.008 (PMC10239778; doi:10.1016/j.ekir.2023.03.008)
Supplement: Supplementary File (PDF) [file mmc1.pdf]

SUPPLEMENTARY MATERIALS

Table S1. Sources and Definitions

Variable	How Defined	Source(s)	Lowest Valid	Highest Valid
Gender	Male or female	Patient-level variable from vendor		
Age	In years	Year of birth; all years before 1928 normalized to 1928 due to privacy concerns		
Race	In Optum-defined categories	Patient-level variables for race and ethnicity from vendor		
Region	U.S. Census Regions	Patient-level variable from vendor		
Baseline serum bicarbonate (mEq/L)	The first of the two consecutive, valid, consistent values (i.e. both 12 to <22 or 22 to 29) separated by 28–365 days that establish index date	Laboratory tests in all settings of care	8	40
Baseline eGFR in mL/min/1.73 m^2	The mean of eGFR values (CKD-EPI) during the 90 days preceding the last eGFR test on or before the index date; multiple values per calendar day were averaged to contribute singly to this mean	Serum creatinine laboratory tests not collected during inpatient hospitalizations or emergency department visits with a diagnosis of acute kidney injury	Serum creatinine >0	Serum creatinine <20
Chronic Kidney Disease				
Stage G3a	eGFR 45 to <60 mL/min/1.73 m^2	Reference: Baseline eGFR, calculated with the 2009 CKD-EPI creatinine equation for adults, KDIGO 2012 Clinical Practice Guideline for the Evaluation and Management of Chronic Kidney Disease. Kidney International Supplements, Vol 3, Issue 1. January 2013.		
Stage G3b	eGFR 30 to <45 mL/min/1.73 m^2			
Stage G4	eGFR 15 to <30 mL/min/1.73 m^2			
Non-dialysis stage G5	eGFR > 10 to <15 mL/min/1.73 m^2			
Comorbidities / conditions				

Variable	How Defined	Source(s)	Lowest Valid	Highest Valid
Diabetes	ICD-9CM: 250; 357.2; 362.0; 366.41; ICD10-CM: E08.311-E08.36; E08.40; E08.42; E09.311-E09.36; E09.40; E09.42; E10.10-E13.9	One or occurrences of an ICD-9-CM or ICD-10-CM diagnosis code in data extracted from medical or administrative data, with a diagnosis date occurring in all available data prior to and not including the index date (minimum, 3 years). Definitions include all extensions. In ICD-10 data, header codes are included if all subordinate codes are included in the definition. For all comorbidities, codes with a diagnosis status of "Possible diagnosis of", "History of", or "Family history of" were excluded.<br><br><u>Reference:</u><br>1) United States Renal Data System 2018 annual data report: Epidemiology of kidney disease in the United States. National Institutes of Health, National Institute of Diabetes and Digestive and Kidney Diseases, Bethesda, MD, 2018: CKD Analytical Methods.		
Hypertension	ICD9-CM: 362.11; 401-405; 437.2; ICD10-CM: H35.031-H35.039, I10-I13.2, I15.0-I15.9, I67.4, N26.2			
Acute kidney injury (AKI)	ICD-9-CM diagnosis code between 584.5 and 584.9 or an ICD-10-CM diagnosis code between N17.0 and N17.9 during dates of a hospital emergency room visit or hospital inpatient or observation admission.	AKI is defined here because eGFR and serum bicarbonate values obtained during hospital inpatient or emergency department care involving AKI are excluded.		
Additional baseline labs				
Urine albumin-creatinine ratio (ACR, mg/g)	Urine protein-to-creatinine ratios were converted to ACR by dividing by 2.655 for women and 1.7566 for men.1 Dipstick urine results were assigned estimated ACR values in mg/g as follows: -, negative = 9; "trace" = 43; Plus, plus one, 1, 1+ or + = 81; Plus 2, 2 plus, 2+ = 315; 3+, 4+ or similar = 1073.	Laboratory value for ACR or any test convertible to ACR occurring closest ≤ index date, using all available data. ACR was log-transformed in regression analyses due to skew.<br><br><u>Reference:</u><br>1) Inker LA, Levey AS, Pandya K, et al. Early change in proteinuria as a surrogate end point for kidney disease		

Variable	How Defined	Source(s)	Lowest Valid	Highest Valid
		progression: an individual patient meta-analysis. Am J Kidney Dis. 2014;64(1):74-85.<br>2) Tangri, JAMA. 2016;315(2):164-174. Supplemental Material e1.2		
Serum albumin (SA, g/dL)	Laboratory value closest ≤ index date	Laboratory value as reported. Imputed if missing.	1	6
Serum calcium, corrected (mg/dL)	Laboratory value closest ≤ index date within 1 year, calculated as [total calcium + 0.8 × (4 – serum albumin)] using baseline serum albumin if available, otherwise as report	Laboratory value as reported. Imputed if missing.	4	18
Kidney Failure Risk Equation (KFRE)				
2- year risk of kidney failure, using 8-variable risk equation, as used	risk= 1-0.9780**exp((-0.1992 * (age/10-7.036) + 0.1602 * (male-0.5642)- 0.4919 * (eGFR/5- 7.222) + 0.3364 * (log_ACR-5.137)-0.3441 * (serum albumin-3.997) + (-0.07354 *(serum bicarbonate-25.57))- 0.2228 * (calcium-9.355)))	Computed using index values. Phosphorous was omitted due to high levels of missing data.<br>Because the equation calculates risk as a decimal percentage, in statistical models we multiplied by 100 so that each 1-unit increment would represent a 1-percentage point increase in risk of kidney failure.	<u>Reference:</u> Tangri N, Stevens LA, Griffith J, Tighiouart H, Djurdjey O, Naimark D, Levin A, Levey AS. A predictive model for progression of chronic kidney disease to kidney failure. JAMA 2011;305(15):1553-1559.	
2- year risk of kidney failure, using 4-variable risk equation	risk= 1-0.9750**exp(-0.2201 * (age/10-7.036) + 0.2467 * (male-0.5642) - 0.5567 * (eGFR/5 - 7.222) + 0.4510 * (log ACR- 5.137))	Computed using index values.<br>Because the equation calculates risk as a decimal percentage, in statistical models we multiplied by 100 so that each 1-unit increment would represent a 1-percentage point increase in risk of kidney failure.		

Variable	How Defined	Source(s)	Lowest Valid	Highest Valid
Outcomes				
Cost	All-cause cost per month for medical services (claims other than pharmacy), inflated from Optum standard cost in 2015 USD to 2021 USD at 3% per annum.	1% winsorization (range ≥0 to 55,642.18. values outside these limits set to limit)		

AKI, acute kidney injury; CKD-EPI, Chronic Kidney Disease Epidemiology Collaboration; CPT, Current Procedural Terminology®; eGFR, estimated glomerular filtration rate; EMR, electronic medical record; HCPCS, Healthcare Common Procedural Coding System; ICD-CM, International Statistical Classification of Diseases and Related Health Problems, Clinical Modification

Table S2. Kidney Failure Risk Equation

8-variable equation, Patient 2-year risk:

**Original**	**1 – 0.9780 ^ exp (-0.1992 × (age/10 – 7.036) + 0.1602 × (male – 0.5642) – 0.4919 × (eGFR/5 – 7.222) + 0.3364 × (logACR – 5.137) – 0.3441 × (albumin – 3.997) + 0.2604 × (phosphorous – 3.916) – 0.07354 × (bicarbonate – 25.57) – 0.2228 × (calcium – 9.355))**
Regional Calibrated Original – North America	1 – 0.9757 ^ exp (-0.1992 × (age/10 – 7.036) + 0.1602 × (male – 0.5642) – 0.4919 × (eGFR/5 – 7.222) + 0.3364 × (logACR – 5.137) – 0.3441 × (albumin – 3.997) + 0.2604 × (phosphorous – 3.916) – 0.07354 × (bicarbonate – 25.57) – 0.2228 × (calcium – 9.355))
**Regional Calibrated Original – non-North America**	**1 – 0.9827 ^ exp (-0.1992 × (age/10 – 7.036) + 0.1602 × (male – 0.5642) – 0.4919 × (eGFR/5 – 7.222) + 0.3364 × (logACR – 5.137) – 0.3441 × (albumin – 3.997) + 0.2604 × (phosphorous – 3.916) – 0.07354 × (bicarbonate – 25.57) – 0.2228 × (calcium – 9.355))**
Pooled	1 – 0.9629 ^ exp (-0.1848 × (age/10 – 7.036) + 0.2906 × (male – 0.5642) – 0.4156 × (eGFR/5 – 7.222) + 0.3480 × (logACR – 5.137) – 0.3569 × (albumin – 3.997) + 0.1582 × (phosphorous – 3.916) – 0.01199 × (bicarbonate – 25.57) – 0.1581 × (calcium – 9.355))

4-variable equation, Patient 2-year risk:

**Original**	**1 – 0.9750 ^ exp (-0.2201 × (age/10 – 7.036) + 0.2467 × (male – 0.5642) – 0.5567 × (eGFR/5 – 7.222) + 0.4510 × (logACR – 5.137))**
Regional Calibrated Original – North America	1 – 0.9751 ^ exp (-0.2201 × (age/10 – 7.036) + 0.2467 × (male – 0.5642) – 0.5567 × (eGFR/5 – 7.222) + 0.4510 × (logACR – 5.137))
**Regional Calibrated Original – non-North America**	**1 – 0.9832 ^ exp (-0.2201 × (age/10 – 7.036) + 0.2467 × (male – 0.5642) – 0.5567 × (eGFR/5 – 7.222) + 0.4510 × (logACR – 5.137))**
Pooled	1 – 0.9676 ^ exp (-0.2245 × (age/10 – 7.036) + 0.3212 × (male – 0.5642) – 0.4553 × (eGFR/5 – 7.222) + 0.4469 × (logACR – 5.137))

Item S1. STROBE Statement

- STROBE Statement—checklist of items that should be included in reports of observational studies
-

	• Item No.	• Recommendation	• Page No.	• Relevant text from manuscript
**Title and abstract**	1	(*a*) Indicate the study's design with a commonly used term in the title or the abstract	1	Association of the Kidney Failure Risk Equation with High Healthcare Costs
		(*b*) Provide in the abstract an informative and balanced summary of what was done and what was found	2	Generalized linear regression models were used to examine the association of KFRE score and healthcare costs.
**Introduction**				
Background/rationale	2	Explain the scientific background and rationale for the investigation being reported	5	Risk as determined by the KFRE and its association with costs in the US health care system, as well as a broader general

				population not referred for nephrology care, have not been studied.
Objectives	3	State specific objectives, including any prespecified hypotheses	5	As such, we aimed to determine the relationship between risk as classified by the KFRE and all-cause medical costs from the perspective of the US health system payer.
**Methods**				
Study design	4	Present key elements of study design early in the paper	6	This observational, retrospective cohort study of U.S. patients with advanced non-dialysis-dependent CKD was conducted using Optum's de-identified Integrated Claims-Clinical dataset of US patients (2007-2017).
Setting	5	Describe the setting, locations, and relevant dates, including periods of	6	The Optum database is a longitudinal repository of electronic health record

		recruitment, exposure, follow-up, and data collection		(EHR) data that included >81 million patients as of 2017 from all insurance types/statuses, and a subset of patients linked by unique patient identifiers to a healthcare claims database of private and Medicare Advantage healthcare insurance plans.[18] The Optum database includes patients from several healthcare provider organizations across all 50 U.S. states and Puerto Rico.
Participants	6	(*a*) *Cohort study*—Give the eligibility criteria, and the sources and methods of selection of participants. Describe methods of follow-up	6-7	This was an ancillary study of a larger observational, retrospective study to determine the association of serum bicarbonate levels with clinical outcomes. Patients included in the database extract with claims data had at

	Case-control study—Give the eligibility criteria, and the sources and methods of case ascertainment and control selection. Give the rationale for the choice of cases and controls<br>*Cross-sectional study*—Give the eligibility criteria, and the sources and methods of selection of participants		least one year of EHR activity with at least 3 estimated glomerular filtration rate (eGFR) results <60 mL/min/1.73 m^2 and at least 3 serum bicarbonate results, with at least one result between 12 and 29 mEq/L, plus linked claims data (**Figure 1**).
	(*b*) *Cohort study*—For matched studies, give matching criteria and number of exposed and unexposed<br>*Case-control study*—For matched studies, give matching criteria and the number of controls per case	N/A	

Variables	7	Clearly define all outcomes, exposures, predictors, potential confounders, and effect modifiers. Give diagnostic criteria, if applicable	7-8	The primary variable of interest was the predicted risk of kidney failure within 2 years, calculated for each patient using the 8-variable and 4-variable versions of the Kidney Failure Risk Equation (KFRE).[11, 12]<br><br>The outcome was all-cause medical costs per month, assessed during the 2-year outcome period. Outpatient pharmacy costs were not included in this analysis. Medical costs in the Optum dataset were obtained from medical insurance claims for inpatient and outpatient services of all types, and provided from the payer perspective

				(i.e., health plan payments rather than provider billed charges) as Optum-defined “standard cost”, an approximated cost per service based on private insurance payment levels in 2015 US dollars (USD).
Data sources/ measurement	8*	For each variable of interest, give sources of data and details of methods of assessment (measurement). Describe comparability of assessment methods if there is more than one group	*6*	Data extracted from inpatient and outpatient EHRs and administrative systems included laboratory results, prescribed medications, coded diagnoses and procedures, and provider notes extracted by natural language processing. Institutional review board oversight was not required. because the Optum EHR Database contained only HIPAA-compliant, de-identified data.

Bias	9	Describe any efforts to address potential sources of bias	N/A	
Study size	10	Explain how the study size was arrived at	6	This observational, retrospective cohort study of U.S. patients with advanced non-dialysis-dependent CKD was conducted using Optum's de-identified Integrated Claims-Clinical dataset of US patients (2007-2017). The Optum database is a longitudinal repository of electronic health record (EHR) data that included >81 million patients as of 2017 from all insurance types/statuses, and a subset of patients linked by unique patient identifiers to a healthcare claims database of private and Medicare Advantage healthcare insurance plans

Quantitative variables	11	Explain how quantitative variables were handled in the analyses. If applicable, describe which groupings were chosen and why	7-8	The primary variable of interest was the predicted risk of kidney failure within 2 years, calculated for each patient using the 8-variable and 4-variable versions of the Kidney Failure Risk Equation (KFRE).[11, 12] The 4-variable KFRE predicts a patient's risk of kidney failure using sex, age, eGFR, and log-transformed urine ACR, while the 8-variable version adds serum albumin, serum bicarbonate, serum calcium and serum phosphorous (**Supplementary Table 2**). In this study, serum bicarbonate and eGFR were evaluated at baseline. Urine ACR was evaluated using the value reported for an available urine ACR or value converted from a urine protein-to-creatinine ratio (PCR) or dipstick urine test[20, 21] that occurred closest to or before the index date within 1 year. Corrected calcium was calculated using baseline

				serum albumin if available, otherwise unadjusted total calcium was used. Data on calcium, phosphorous and albumin was limited to values occurring within a year on or before the index date.
Statistical methods	12	(*a*) Describe all statistical methods, including those used to control for confounding	10	GLM models were run within CKD stage G3 and stage G4 groups, equating log transformed medical cost per month with the calculated risk score with both the 8-variable and 4-variable KFREs. Since the 8-variable KFRE risk score models contained missing serum calcium and serum albumin data, multiple imputation was implemented 10 times to generate 10 complete datasets. These 10 complete datasets were then analyzed separately using the 8-variable KFRE risk score as the sole predictor of medical cost per month (log-transformed). The results of the 10 analyses were

		combined in the SAS PROC MIANALYZE procedure to derive valid inferences. Similar sets of GLM analyses were run on models that evaluated each component of the 4- and 8-variable KFRE equations as individual covariates: 1) a model with age, sex, eGFR, and log-transformed urine ACR (4 variable); 2) a model with age, sex, eGFR, log-transformed urine ACR, serum bicarbonate, serum albumin, and serum calcium (8 variable, excluding serum phosphorous).
(*b*) Describe any methods used to examine subgroups and interactions	10	GLM models were run within CKD stage G3 and stage G4 groups
(*c*) Explain how missing data were addressed	10	Since the 8-variable KFRE risk score models contained missing serum calcium and serum albumin data, multiple imputation was implemented 10 times to generate 10 complete datasets. These 10 complete datasets were

			then analyzed separately using the 8-variable KFRE risk score as the sole predictor of medical cost per month (log-transformed). The results of the 10 analyses were combined in the SAS PROC MIANALYZE procedure to derive valid inferences.
	(*d*) *Cohort study*—If applicable, explain how loss to follow-up was addressed<br>*Case-control study*—If applicable, explain how matching of cases and controls was addressed<br>*Cross-sectional study*—If applicable, describe analytical methods taking account of sampling strategy	7, Figure 1	Study inclusion also required EHR activity for ≥6 months pre-index plus ≥2 years post-index unless the patient died within this 2-year period.
	(*e*) Describe any sensitivity analyses	10	Similar sets of GLM analyses were run on models that evaluated each component of the 4- and 8-variable

				KFRE equations as individual covariates: 1) a model with age, sex, eGFR, and log-transformed urine ACR (4 variable); 2) a model with age, sex, eGFR, log-transformed urine ACR, serum bicarbonate, serum albumin, and serum calcium (8 variable, excluding serum phosphorous).
**Results**				
Participants	13*	(a) Report numbers of individuals at each stage of study—eg numbers potentially eligible, examined for eligibility, confirmed eligible, included in the study, completing follow-up, and analysed	11, Figure 1	The Optum database contained longitudinal records on 81 million patients, of which 29,831 met the criteria for inclusion in the database extract with claims. Within this extract, 1,721 patients qualified for the study cohort: 1,475 in CKD stage G3 and 246 in CKD stage G4 (**Figure 1**).
		(b) Give reasons for non-participation at each stage	Figure 1	See figure 1

		(c) Consider use of a flow diagram	Figure 1	See figure 1
Descriptive data	14*	(a) Give characteristics of study participants (eg demographic, clinical, social) and information on exposures and potential confounders	11, Table 1	Mean (standard deviation [SD]) age was 75.3 (9.8) years in the CKD stage G3 group and 76.2 (9.1) years in the CKD stage G4 group (**Table 1**). The CKD stage G3 and G4 groups were 49% and 46% male, 88% and 85% White race, respectively. Mean (SD) baseline serum bicarbonate was 25.5 (4.3) mEq/L in patients with CKD stage G3 and 23.2 (4.6) mEq/L in the CKD stage G4, while mean (SD) serum albumin was 3.8 (0.6) g/dL and 3.6 (0.6) g/dL, respectively. Diabetes was identified in 39% of the CKD stage G3 group and 50% of the CKD stage G4 group. Hypertension was highly prevalent in both groups; 73% in CKD stage G3 and 80% in CKD stage G4. Kidney failure risk scores using the 4- and 8-variable KFRE equations showed an average predicted risk of kidney failure within the next 2 years of 0.8%-

				1.0% in the CKD stage G3 group and 8.6%-9.2% in the CKD stage G4 group, respectively.
		(b) Indicate number of participants with missing data for each variable of interest	8	Serum calcium was missing in 5% of patients in CKD stage 3, and 0% of patients in CKD Stage 4, and serum albumin was missing in 21% of patients in CKD stage 3, and 13% of patients in CKD stage 4.
		(c) *Cohort study*—Summarise follow-up time (eg, average and total amount)	Figure 1	Required 2 year follow up or death – Figure 1
Outcome data	15*	*Cohort study*—Report numbers of outcome events or summary measures over time	*11-12, Figure 2 and Figure 3*	Patients in the highest quartile of kidney failure risk as measured by the 8-variable KFRE had average costs per month over a 2-year outcome period of $8,765 in stage G3a, $11,424 in stage G3b, and $12,006 in stage G4, vs $6,021 (*P* = 0.003), $7,700 (*P* = 0.002) and $11,505 (*P* = 0.8) for patients in the second-highest quartile of kidney failure risk, respectively (**Figure 2**). More advanced

CKD was consistently associated with higher costs. Average cost per month was approximately double between the lowest and highest quartile for patients with CKD stage G3a ($8,765 vs. $4,125), and approximately 2.5 to 3-fold higher between the lowest and highest quartile in patients with CKD stage G3b ($11,424 vs. $4,455) and in patients with CKD stage G4 ($12,006 vs. $4,827). In all CKD stages, costs were statistically similar between the two lowest quartiles of kidney failure risk (**Figure 2**). When considering the entire population not stratified into CKD stages, patients in the highest quartile of risk had a monthly cost of $10,934, over 2.5-fold higher than those in the lowest risk quartile with a monthly cost of $4,000. Those in the middle quartiles were not statistically different from each other

				with monthly costs ranging from $5,989 to $6,744 (**Figure 3**).
		Case-control study—Report numbers in each exposure category, or summary measures of exposure	*N/A*	
		Cross-sectional study—Report numbers of outcome events or summary measures	*N/A*	
Main results	16	(*a*) Give unadjusted estimates and, if applicable, confounder-adjusted estimates and their precision (eg, 95% confidence interval). Make clear which confounders were adjusted for and why they were included	12	The 8-variable KFRE was found to be significantly associated with healthcare costs in patients with CKD stage G3 and G4. Adjusted analyses indicated that each 1% (absolute) increase in the predicted risk score was associated with a 13.5% (parameter estimate 0.126, 95% confidence interval [CI]: 0.085-0.167; *P* < 0.0001) and 4.1% (parameter estimate 0.040, 95% CI: 0.022 to 0.058,

			P < 0.0001) increase in predicted monthly healthcare costs in patients with CKD stages G3 and G4, respectively (**Table 2**). The fully specified model in patients with CKD stage G3 found 2 variables to be significant predictors of monthly costs: 1) serum bicarbonate (1 mEq/L increase associated with a 7.0% decrease in monthly costs); 2) serum albumin (1 g/dL increase associated with a 55.5% decrease in monthly costs). These findings were similar among patients with CKD stage G4: 1) serum bicarbonate (1 mEq/L increase associated with a 4.6% decrease in monthly costs); 2) serum albumin (1 g/dL increase associated with a 52.1% decrease in monthly costs).
	(*b*) Report category boundaries when continuous variables were categorized	12	

	(*c*) If relevant, consider translating estimates of relative risk into absolute risk for a meaningful time period	N/A	

Other analyses	17	Report other analyses done—eg analyses of subgroups and interactions, and sensitivity analyses	N/A	
**Discussion**				
Key results	18	Summarise key results with reference to study objectives	14	In this study of patients with CKD stages G3-G4, we found that higher risk of progression to kidney failure as calculated by the KFRE was associated with higher monthly all-cause medical costs in a population-based sample of US patients. These findings are consistent with previous Canadian data associating KFRE based risk to healthcare costs,[17] and extend these observations to a larger dataset in the US health care system. As nephrology practices integrate the KFRE in their electronic health records or clinical decision support software, they can assume that the highest risk individuals as identified by the equation are also likely to have the highest health care costs.

Limitations	19	Discuss limitations of the study, taking into account sources of potential bias or imprecision. Discuss both direction and magnitude of any potential bias	15-16	This analysis also had several limitations. First, the cost data was obtained from patients with two types of common insurance (private and Medicare Advantage) and may not be representative of other payment types (e.g., Medicaid). Urine ACR and serum bicarbonate were required for inclusion in the study for assessment of the KFRE (measured directly or by transforming a urine PCR/urine dipstick result), and as such, may not be representative of patients in whom no such measurement was available. While these findings confirm a previous study in the Canadian health system,[17] they may not reflect implications, risks, and costs in other countries; these country-specific analyses may require future evaluation. In addition, as outpatient pharmacy costs were not available in the Optum dataset, we were unable to include them in our results. Lastly, we were unable to study the effects of

				unmeasured variables such as detailed information on socioeconomic health status for which data were not available.
Interpretation	20	Give a cautious overall interpretation of results considering objectives, limitations, multiplicity of analyses, results from similar studies, and other relevant evidence	16	In conclusion, higher risk of progression to kidney failure as measured by the KFRE is associated with higher all-cause health care costs in patients with private insurance and Medicare Advantage in the United States. Use of risk stratification equations to identify patients at the highest risk of progression, and subsequent use of health care resources may help to effectively target resources.
Generalisability	21	Discuss the generalisability (external validity) of the study results	14	To our knowledge, this analysis is the first to evaluate the association of the risk of CKD progression as measured by the KFRE and associated health care costs in a US population. A previous study was performed in Canada among patients referred to a multidisciplinary care CKD clinic that examined

5-year costs and their association with risk of CKD progression as measured by the KFRE and found that the total annual cost of hospital admissions, physician visits, and drug dispensations totaled over $134,000 for patients at high risk of progression (>15% risk of failure over 5 years) versus approximately $76,000 for those at low risk of progression (Canadian dollars).[17] Our study differed from the Canadian study as it was done in a US population and included all identified patients with CKD in Optum rather than those enrolled in a multidisciplinary clinic with subjective enrollment criteria. A previous study of private and Medicare patients in the US found the annual cost of non-dialysis CKD care to range between $26,000 and $77,000 for patients with CKD stages G3a-G5 with private insurance coverage, and between $21,000 and $46,000 for patients receiving Medicare (2016 USD).[7]

**Other information**				
Funding	22	Give the source of funding and the role of the funders for the present study and, if applicable, for the original study on which the present article is based	17	The authors would like to thank Dawn Parsell and Jun Shao (both employees of Tricida) for review of the manuscript and help with the design of figures. Editorial support was provided by Jun Shao.<br>This study was funded by Tricida, Inc.

*Give information separately for cases and controls in case-control studies and, if applicable, for exposed and unexposed groups in cohort and cross-sectional studies.

Note: An Explanation and Elaboration article discusses each checklist item and gives methodological background and published examples of transparent reporting. The STROBE checklist is best used in conjunction with this article (freely available on the Web sites of PLoS Medicine at http://www.plosmedicine.org/, Annals of Internal Medicine at http://www.annals.org/, and Epidemiology at http://www.epidem.com/). Information on the STROBE Initiative is available at www.strobe-statement.org.
